# A/T Run Geometry of B-form DNA Is Independent of Bound Methyl-CpG Binding Domain, Cytosine Methylation and Flanking Sequence

**DOI:** 10.1038/srep31210

**Published:** 2016-08-09

**Authors:** Jyh Yea Chia, Wen Siang Tan, Chyan Leong Ng, Nien-Jen Hu, Hooi Ling Foo, Kok Lian Ho

**Affiliations:** 1 Department of Pathology, Faculty of Medicine and Health Sciences; Universiti Putra Malaysia, 43400 UPM Serdang, Selangor, Malaysia; 2 Department of Microbiology, Faculty of Biotechnology and Biomolecular Sciences; Universiti Putra Malaysia, 43400 UPM Serdang, Selangor, Malaysia; 3 Institute of Bioscience, Universiti Putra Malaysia, 43400 UPM Serdang, Selangor, Malaysia; 4 Institute of Systems Biology, Universiti Kebangsaan Malaysia, 43600 UKM Bangi, Selangor, Malaysia; 5 Institute of Biochemistry, National Chung Hsing University, 250, Guoguang Rd., Taichung City 402, Taiwan; 6 Department of Bioprocess Technology, Faculty of Biotechnology and Biomolecular Sciences; Universiti Putra Malaysia, 43400 UPM Serdang, Selangor, Malaysia

## Abstract

DNA methylation in a CpG context can be recognised by methyl-CpG binding protein 2 (MeCP2) via its methyl-CpG binding domain (MBD). An A/T run next to a methyl-CpG maximises the binding of MeCP2 to the methylated DNA. The A/T run characteristics are reported here with an X-ray structure of MBD A140V in complex with methylated DNA. The A/T run geometry was found to be strongly stabilised by a string of conserved water molecules regardless of its flanking nucleotide sequences, DNA methylation and bound MBD. New water molecules were found to stabilise the Rett syndrome-related E137, whose carboxylate group is salt bridged to R133. A structural comparison showed no difference between the wild type and MBD A140V. However, differential scanning calorimetry showed that the melting temperature of A140V constructs in complex with methylated DNA was reduced by ~7 °C, although circular dichroism showed no changes in the secondary structure content for A140V. A band shift analysis demonstrated that the larger fragment of MeCP2 (A140V) containing the transcriptional repression domain (TRD) destabilises the DNA binding. These results suggest that the solution structure of MBD A140V may differ from the wild-type MBD although no changes in the biochemical properties of X-ray A140V were observed.

Methylation at the 5^th^ position of the cytosine pyrimidine ring in a CpG context (methyl-CpG) plays various roles in mammals, including genomic imprinting, gene regulations, X-inactivation, DNA replication and DNA mismatched repair^1^. Methyl-CpG dinucleotides can be recognised by a family of proteins sharing a common, but not identical, methyl-CpG binding domain (MBD)^2^. To date, a total of 5 family members, namely MeCP2, MBD1, MBD2, MBD3 and MBD4, have been identified based on their amino acid sequence similarity^2^. Functionally, the MBD domains of the family members bind the methyl-CpG dinucleotide of the methylated DNA, except for mammalian MBD3, due to substitutions of amino acids that are critical for methyl group recognition^2^. MeCP2, an abundant nuclear protein, is the prototype of the MBD family^3^. The full-length MeCP2 comprises several functional domains. The MBD domain is located between amino acids 78–163 at the N-terminal region, and a transcriptional repression domain (TRD) spanning from amino acids 207–310 is located at the C-terminal region^4^. Upon binding to the methylated DNA via the MBD domain, the TRD domain recruits transcriptional co-repressors such as mSin3A and histone deacetylase I and II (HDACs) for deacetylation and chromatin compaction^4^. Furthermore, MeCP2 has recently been shown to interact with non-CpG methylated cytosine in differentiated neurons^5^,^6^. Moreover, the binding of MeCP2 to 5-hydroxymethylcytosine was also demonstrated to up-regulate the associated gene expression^7^. A nuclear localisation signal (NLS) is found to overlap with the TRD domain between amino acids 255–271. The NLS is responsible for translocating MeCP2 into the cell nucleus^8^. In addition, MeCP2 contains two AT hooks bearing the amino acid sequences ^185^GRGRGRP^191^ and ^265^PKKRGRKP^272^ (underlined superscript indicates amino acid number)^9^,^10^. The amino acid sequence of the AT hooks of MeCP2 is highly similar to the sequence of the high-mobility group with AT hook I chromosomal protein (HMGA-I) that can bind to the minor groove of the A/T stretches (A/T run) of DNA and functionally, perhaps in concert with other chromatin modifiers, leads to chromatin compaction and gene repression^11^,^12^. Zoghbi and colleagues^13^ demonstrated that truncation at R270 of MeCP2, which disrupts the second conserved AT hook of MeCP2, led to failure in the chromatin compaction and localisation of the pericentric heterochromatin domain of the chromatin remodelling protein α-thalassemia mental retardation syndrome X-linked (ATRX). R270X mice were further shown to exhibit Rett syndrome (RTT) phenotypes similar to MeCP2 knock-out mice^13^. RTT is due to mutations in the *MECP2* gene, which result in a progressive neurodevelopmental disorder in early childhood and cause mental retardation in females, with a prevalence of 1 in 10,000–15,000 births^14^. RTT is caused by an X-linked mutation with dominant inheritance, which is normally lethal in males due to severe encephalopathy^15^. In 2005, Bird and colleagues^16^ showed that an A/T run adjacent to the methyl-CpG is required to enhance the MeCP2 binding. The requirement of an A/T run next to the methyl-CpG dinucleotide has not been reported in other MBD proteins. MBD1 preferentially binds T^m^CGCA and TG^m^CGCA (^m^C represents 5-methyl-cytosine) sequences^17^. The sequence specificity of MBD2 outside the methyl-CpG motif has not been conclusively defined, although a guanine following methyl-CpG (^m^CpGG) was reported to enhance MBD binding^18^. On the other hand, MBD4 recognises the G/T mismatch of ^m^CpG/TpG and ^m^CpG/CpG (hemimethylated-CpG) in addition to the methyl-CpG dinucleotide^19^. The C-terminal G/T mismatch DNA glycosylase of MBD4 is then involved in DNA mismatch repair^19^,^20^. MBD4 has also been demonstrated to recognise diverse modifications at the 5^th^ position of pyrimidine, such as 5-formylcytosine, 5-carboxycytosine and 5-hydroxymethyluracil^21^. Additionally, MBD4 has been shown to function as a transcriptional repressor^20^.

In the context of an A/T run, known endogenous MeCP2 targeting genes such as the mouse *brain derived neurotrophic factor* (*BDNF*) promoter region contain high occurrences of A/T runs close to the methyl-CpGs^22^,^23^. Nonetheless, the characteristics of the A/T run that provide the specificity for MeCP2 to recognise the methyl groups in a methyl-CpG context remained unknown. There is also the question of whether the binding of the MBD domain would affect the A/T run geometry or *vice versa*. Due to the presence of AT hooks in MeCP2 and the requirement of the A/T run for maximal binding, it has been speculated that the A/T run could interact with the AT hooks of MeCP2. Our aim in this study was to elucidate the characteristics of the A/T run with the MBD domain bound to its adjacent methyl-CpG. Together with the hypothesis that the A/T run might interact with the AT hook of MeCP2, we report here the geometry of the A/T run adjacent to the methyl-CpG dinucleotide in the presence of bound MeCP2 MBD domain, together with the structure of the MBD A140V mutant (MBD^A140V^). The oligonucleotide sequence used in this study was derived from nucleotides −108 to −90 of the mouse *BDNF* promoter III region (*BDNF*^*ProIII*^). MeCP2 A140V is a non-classical RTT mutant that mainly affects males, with abnormalities of cell packing density and reduced dendritic branching of cortical pyramidal neurons^24^,^25^,^26^. In this study, we used the pathologically important MeCP2 A140V mutant to prepare crystals of MBD^A140V^ in complex with the methylated *BDNF*^*ProIII*^. The co-crystals diffracted X-rays to 2.2 Å resolution at a wavelength of 0.9795 Å. This higher resolution co-crystal structure provides more insight than the previously reported X-ray structure by Ho *et al*.^27^. The atomic details of the DNA geometry of the MBD-bound A/T run are highlighted in comparison with the A/T run of the free DNA double helices and also the DNA in complex with the AT hook of HMGA-1. The results of this study revealed that the A/T run geometry of the methylated DNA is not affected by the bound MeCP2 MBD domain or the flanking nucleotide sequences.

## Results and Discussion

### A140V mutation destabilises the MeCP2-methylated DNA interaction

**Figure 1:**
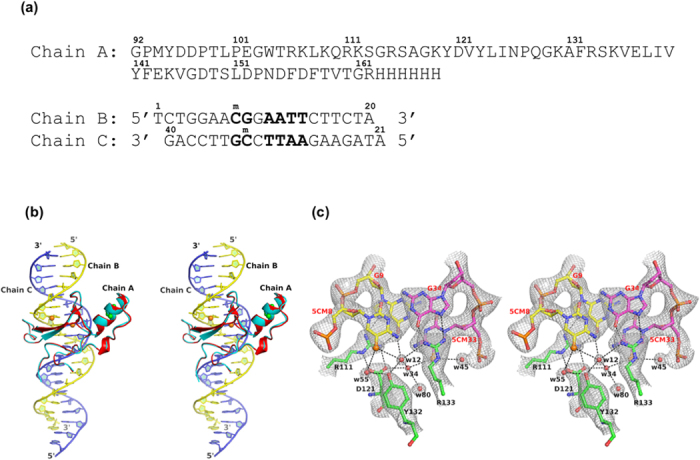
Overall structure of the MeCP2 MBD^A140V^ in complex with methylated DNA. (**a**) Chain A shows the primary amino acid sequence of the MeCP2 MBD^A140V^ construct used in this study. Chains B and C show the oligonucleotides used in this study. ^m^C represents 5′methyl-cytosine. The 5′ to 3′ direction of the DNA strands and the numbers of the nucleotides are indicated. (**b**) X-ray co-crystal structure of MBD^A140V^ in complex with methylated DNA. The MBD^A140V^ domain (coloured in red) is superimposed with the previously determined X-ray MBD domain (coloured in cyan). The RMSD of the superimposition is 0.26 Å. Chains A, B and C represent the MBD domain and the upper and lower oligonucleotide, respectively, as shown in Fig. 1a. The 5′ to 3′ direction of the DNA strands is indicated. Point mutation A140V is represented by green spheres in the middle of the α-helix. (**c**) Several water molecules (red spheres) mediate the recognition of C5 methyl groups (orange spheres) at the DNA-protein contact interface. The hydrogen bonds involving the water molecules are represented by black dashed lines. The guanidinium groups of R111 and R133, which are held directly above the C5 methyl groups, are hydrogen bonded to the guanine bases of G9 and G34, respectively. The 2*f*_*o*_*-f*_*c*_ electron density map covering the 5C methyl group recognition region is contoured at sigma level 1.6. All residues are labelled appropriately.

**Figure 2:**
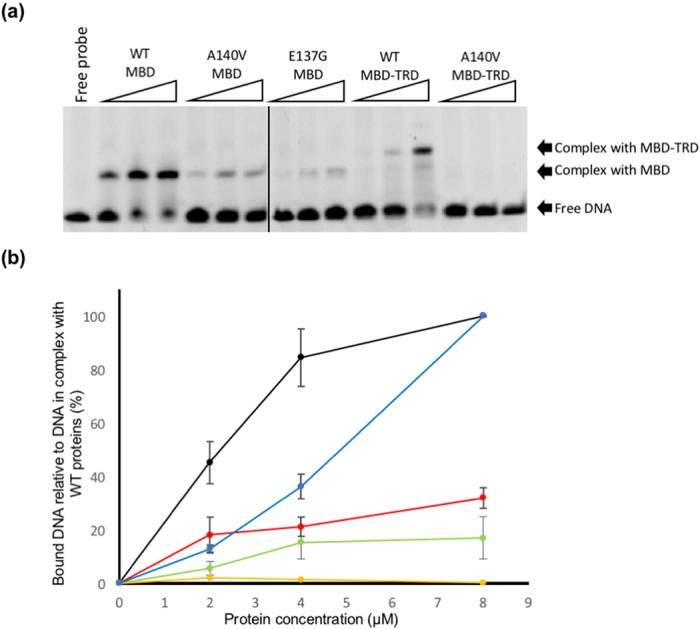
Electrophoretic mobility shift assay. (**a**) The electrophoretic mobility shift assay (EMSA) was performed with the methylated DNA in the presence of wild-type or mutant forms of the MeCP2 MBD and MBD-TRD domains. Each MeCP2 construct was analysed at three different protein concentrations (2 μM, 4 μM, 8 μM). The positions of the methylated DNA, free and in complex with the MBD and MBD-TRD domains, are indicated by arrows. (**b**) A plot of the fraction of the band intensity of the DNA-protein complex at protein concentrations of 2 μM, 4 μM and 8 μM as measured by the Quantity One software. Standard deviations (represented by vertical bars) were calculated from the measured intensities from three separate gels. The values of the band intensity were compared to the intensity of the complex of wild-type MBD or MBD-TRD and the methylated DNA, which was taken as 100%. Plots represent wild-type MBD (Black), MBD^A140V^ (Red), MBD^E137G^ (Green), wild-type MBD-TRD (Blue) and MBD^A140V^-TRD (yellow).

MBD^A140V^ containing a 6xHis tag at its C-terminus was expressed, purified and co-crystallised with a 20-mer double helical B-form DNA (Fig. 1a). The methylated *BDNF*^*ProIII*^ duplex contains a central pair of methyl-CpG dinucleotides and an adjacent A/T run. The co-crystals of the MBD^A140V^-methylated DNA complex diffracted X-rays to 2.2 Å resolution at a wavelength of 0.9795 Å from the synchrotron radiation source at Beamline I02, Diamond Light Source, UK and have unit cell dimensions of *a* = 78.7 Å, *b* = 53.5 Å and *c* = 65.8 Å, with space group C2 (Table 1). The final model shown in Fig. 1b has been refined to final *R*_*cryst*_ and *R*_*free*_ factors of 17.5 and 22.7%, respectively, with a total of 10,482 unique reflections. The overall structure of MBD^A140V^ in complex with the methylated *BDNF*^*ProIII*^ was found to be identical to the previously reported X-ray structure^27^. Nevertheless, the higher resolution of the MBD^A140V^-methylated DNA complex has revealed previously unknown molecular details of various aspects, particularly the DNA geometry, the unique hydration pattern along the minor groove of the DNA molecule and a 3_10_ helix adopted by amino acids ^152^PND^154^ (Fig. 1b). Superimposing MBD^A140V^ and the MBD domain obtained from the previous structure (PDB code 3C2I) using Cα atoms showed an RMSD value of 0.26 Å, indicating that the two structures are identical (Fig. 1b). MeCP2 A140V is a non-classical RTT mutant that mainly affects males and leads to abnormalities of cell packing density and dendritic branching in neurons^24^,^25^. However, one case of MeCP2 A140V-associated disorder with intellectual disability and neuropsychiatric features in a female has also been reported^26^. The A140V mutation has been linked to the disruption of the interaction between MeCP2 and the helicase domain of ATRX *in vitro* and *in vivo* despite the lack of discernible effect of targeting MeCP2 to the methylated DNA^28^. This study revealed that the α-helical region (amino acids 135–145) of the MBD domain is not altered by the A140V mutation. Similarly to previous reports^27^, the C5 methyl groups of bases 5CM8 and 5CM33 are recognised by the MeCP2 MBD domain via several ordered water molecules at the DNA-protein contact interface, and the guanine bases of G9 and G34 are also held in place by two arginine fingers of R111 and R133, respectively (Fig. 1c). To further understand the DNA binding and the secondary structure properties of the A140V mutant and wild-type protein in solution, we have conducted electrophoretic mobility shift assay (EMSA), differential scanning calorimetry (DSC) and circular dichroism (CD) experiments. Although the mutant and wild type proteins are identical according to X-ray observations, to our surprise, the MBD^A140V^ shows reduced complex formation with the methylated DNA compared to the wild-type MBD in the EMSA analysis (Fig. 2a). Quantification of DNA binding showed that MBD^A140V^ binding to methylated DNA is ~68% weaker than the binding of the wild-type MBD (Fig. 2b). CD analysis showed that the wild-type MBD alone consists of ~15 and 45% α-helices and β-strands, respectively (Table 2). The A140V mutation, however, reduces the α-helical regions to ~12% but increases the β-sheet content to ~49%. Interestingly, the total secondary structure contents of both the wild-type MBD and MBD^A140V^ increased by ~10 and 8%, respectively, upon binding to methylated DNA. In both cases, the result clearly indicates that the MBD domain is stabilised by bound methylated DNA. The thermal unfolding parameters of DSC showed that the melting temperature (*T*_*m*_) of MBD^A140V^ in complex with methylated DNA is 7° lower than for the wild-type MBD in complex with methylated DNA (Table 2). Although the X-ray analysis suggests that MBD^A140V^ and wild-type MBD in complex with methylated DNA are identical, both the EMSA and CD analyses suggest that the solution structures of wild-type MBD and A140V mutant protein of MeCP2 are indeed different. DSC analysis further indicates that the A140V mutation destabilises the MBD domain.

To analyse the effect of the A140V mutation on the TRD domain, EMSA analyses with larger fragments of MeCP2 comprising the MBD and TRD domains were conducted. The result showed that the MBD^A140V^-TRD mutant failed to shift the methylated DNA on a native gel compared to the wild-type MBD-TRD, indicating that the interaction of MBD^A140V^-TRD and methylated DNA is interrupted by A140V mutation (Fig. 2a,b). Furthermore, DSC analysis of the MBD^A140V^-TRD in complex with methylated DNA showed a similar trend to MBD^A140V^: the complex was found to have a melting temperature 7° lower than its wild type counterpart (Table 2 and Supplementary Fig. S2). Overall, the band shift and DSC analyses suggest that the A140V mutation may destabilise the inter-domain interactions of MeCP2.

### A/T run displays narrow minor groove of DNA

**Figure 3:**
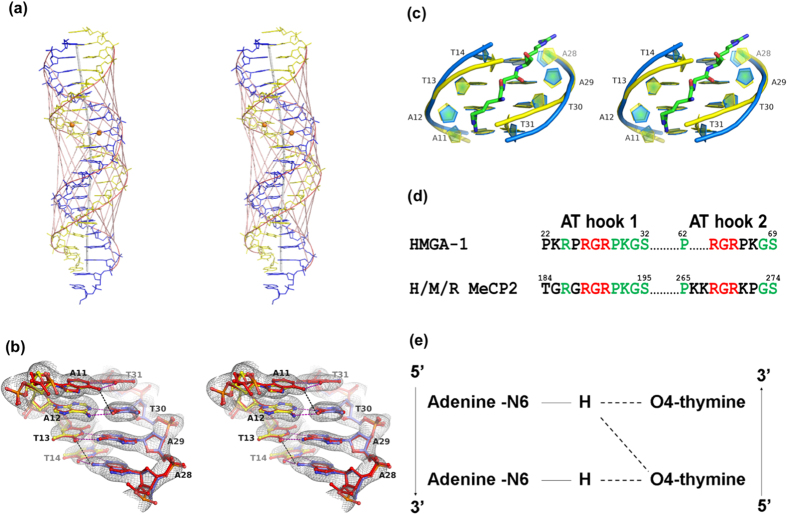
High degree of propeller twist at A/T run base-pairs. (**a**) Schematic drawing of the DNA helix with all the minor and major groove distances generated by *CURVES+*. The DNA backbones are shown in yellow (Chain B) and blue (Chain C), as in Fig. 1b. Inter-phosphate distances across the minor and major grooves are represented by vectors coloured in purple. The calculated helical axis in the centre of the DNA double helix is represented by a dark grey line. (**b**) Stereo view of the major groove formed by ^11^AATT^14^/^28^AATT^31^ base-pairs. The purple and black dashes represent the conventional Watson-Crick hydrogen bonds between adenine-thymine base-pairs and the non-Watson-Crick hydrogen bonds diagonally across the major groove of the A/T run, respectively. All bases are labelled appropriately. Yellow and blue oligonucleotides represent chains B and C, respectively, as shown in Fig. 1. The 2*f*_*o*_*-f*_*c*_ electron density map covers all the bases when contoured at sigma level 1.6 (**c**) Superimposition of A/T run in this study (yellow) and in complex with HMGA-I (blue) gave an RMSD of 1.15 Å. The AT hook motif RGR is shown as a stick model coloured in green, inserted into the minor groove of the A/T run. (**d**) Protein sequence alignment of HMGA-1 AT hooks with MeCP2 AT hook 1 (amino acids 184–195) and AT hook 2 (amino acids 265–274) derived from human (H), mouse (M) and rat (R). The conserved RGR motif, which is speculated to be inserted into the minor groove of A/T run, is coloured in red. (**e**) A zig-zag conformation of the base-pair step resulting from cross-strand non-Watson-Crick hydrogen bonding. The direction of the DNA strands is indicated from 5′ to 3′.

The full-length MeCP2 contains 2 conserved AT hook domains, although Baker *et al*.^13^ argued that a third AT hook preceded the endogenous 6xHis tag of MeCP2. The AT hook-2 domain (amino acids 265–273) of MeCP2 has been shown to be associated with chromatin compaction activities, and mutation of this domain caused RTT-like phenotypes in mice^13^. The AT hook amino acid sequence of MeCP2 is highly similar to the AT hook amino acid sequence of HMGA-I, a non-histone chromatin associated protein that interacts with an A/T run of DNA double helix, as demonstrated by Reeves and Nissen^12^. Based on the similarity of the AT hook sequence, the AT hooks of MeCP2 are also likely to interact with the A/T rich region of the DNA. Figure 3a shows a schematic drawing of the 20-mer DNA duplex observed in this crystal structure. All the inter-phosphate distances across the minor and major grooves of the DNA duplex are drawn. The oligonucleotides annealed to form a Watson-Crick double stranded DNA with 2 complete helical turns. Local base-pair analysis shows that the DNA is a right-handed B-form duplex with a mean helical twist of 36.3°, an average tilt of −0.1° and an average rise per residue of 3.28 Å (Table 3). The central vertical axis of the DNA shows that the duplex is slightly bent after the methyl-CpG dinucleotide (Fig. 3a). The A/T run (^11^AATT^14^/^28^AATT^31^) shows a narrowed minor groove with an average inter-phosphate distance of 9.2 Å (Fig. 3b, Table 3). The narrowest dinucleotide step with the shortest distance of 8.7 Å was observed at the centre base-pair step (A12T13/A29T30) of the A/T run. The inter-phosphate distances for other base-pair steps are larger than 9.5 Å. In contrast to the minor groove of the A/T run, the inter-phosphate distance across the major groove is widened, with a maximum distance of 20 Å across base-pair step A12T13/A29T30 (Table 3). Superimposition of the A/T run bases of *BDNF*^*ProIII*^ with the ones in a palindromic DNA duplex [d(CGCGAATTCGCG)]_2_, also known as the Dickerson-Drew dodecamer (PDB code: 1BNA)^29^, shows that the geometry of the two A/T runs is identical with an RMSD value of 0.267 Å (Fig. 3b), even though the flanking nucleotide sequences are different. The A/T run in the methylated *BDNF*^*ProIII*^ helix is flanked by ^m^C8G9G10/C32^m^C33G34 and ^15^CTTCTA^20^/^22^TAGAAG^27^ preceding and following the A/T run, respectively, whereas in the Dickerson-Drew dodecamer, the flanking sequences are palindromic CGCG tetranucleotides on both sides of the A/T run. The result indicates that the A/T run geometry remained unchanged regardless of the flanking nucleotide sequences, the methylation of cytosine−8 and −33 of *BDNF*^*ProIII*^ and the bound MBD domain. Earlier, a co-crystal structure of HMGA-1 in complex with a palindromic DNA duplex bearing the nucleotide sequence d(CGAATTAATTCG)_2_ (PDB code 3UXW) showed that the minor groove width of the A/T run (underlined) is enlarged by the presence of the AT hook-bearing amino acid sequence RKP**RGRPK**K^30^. Superimposition of the HMGA-1 AT hook bound A/T run with the A/T run of methylated *BDNF*^*ProIII*^ (RMSD of 1.15 Å) clearly demonstrates that the A/T run minor groove width could be enlarged by the insertion of an AT hook (Fig. 3c). The minimum conserved residues RGR of the AT hook were observed to dehydrate the spine of the A/T run minor groove upon binding^30^. The sequence alignment of the MeCP2 and HMGA-1 AT hooks (Fig. 3d) further shows that MeCP2 contains two conserved **RGR(P/K)** motifs that are similar to HMGA-1, indicating that the AT hooks of MeCP2 may also bind to the minor groove of the A/T run of the methylated DNA, similarly to HMGA-1.

Fonfría-Subirós *et al*.^30^ showed that enlargement of the A/T run minor groove width by a bound AT hook could induce DNA bending, which strongly supports the idea that MeCP2 is also involved in DNA bending and chromatin structure alteration. Structure analysis of MBD^A140V^-methylated *BDNF*^*ProIII*^ complex indeed revealed that a kink is found at nucleotide step G10A11/T31C32, immediately after the methyl-CpG dinucleotide, although the A/T run itself is a relatively straight DNA segment. A previous report has shown that a dodecamer containing a homopolymeric run of six A/T base-pairs (poly-dA/poly-dT), exhibiting nearly zero values for slide, roll and tilt, forms a straight stretch of the helix^31^. The helix of methylated *BDNF*^*ProIII*^ also demonstrated a mean value of almost zero, that is, 0.22°, −0.24°, and −0.16° for slide, roll and tilt, respectively. However, these values are slightly higher at step G10A11/T31C32, measured as 1.07°, 1.72° and −3.45°, respectively (Table 3). It is likely that these geometrical deviations are the main cause for the observed helical bending. As a result, the methylated *BDNF*^*ProIII*^ helix is bent approximately 17° due to a kink at the base-pair step G10A11/C31T32, compared to a 24° bend observed in the complex structure of the AT hook and A/T run containing DNA (PBD code: 3UXW).

### A/T run is stabilised by a high degree of propeller twist and purine-purine stacking

**Figure 4:**
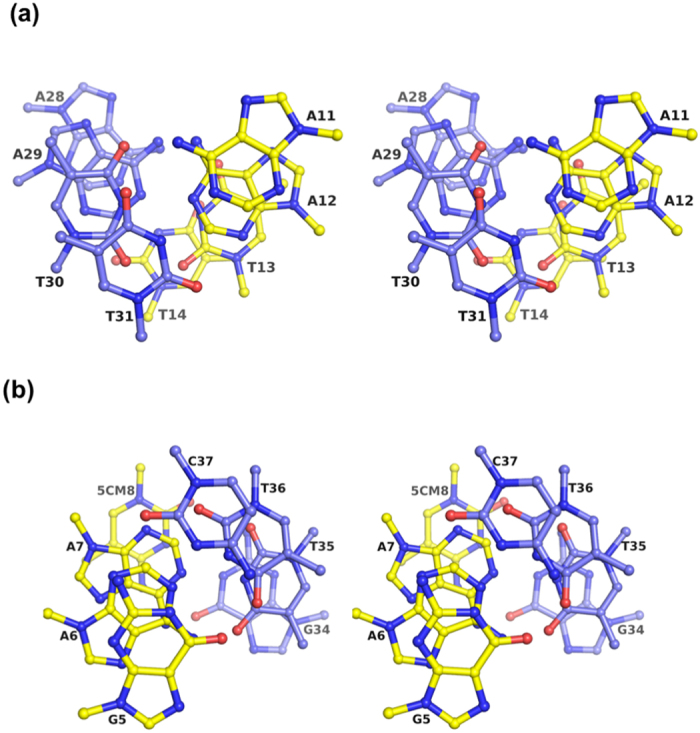
Heavy purine-purine stacking at A/T run base-pair steps. (**a**) Stereo view of ^11^AATT^14^/^28^AATT^31^ base steps looking down the helix from base-pair A11/T31 of the A/T run. (**b**) Stereo view of G5A6A7^m^C8/G34T35T36C37 base-pair steps looking down the helix from base-pair G5/C37. Yellow and blue oligonucleotides represent chains B and C, respectively, as shown in Fig. 1b.

One of the most prominent stabilising forces in the A/T run is the high degree of propeller twist, defined as the rotation of the bases along their longitudinal axis^32^. High propeller twist is characterised by non-coplanarity of the base-pairs, as shown in Fig. 3b. The base-pair propeller twists of this study are tabulated in Table 4. The A/T run base-pairs of the methylated *BDNF*^*ProIII*^ helix are highly propeller twisted with an average of 14.1°, although this value is approximately 3° lower than in the A/T run observed in the Drew-Dickerson dodecamer. In fact, all A/T base-pairs downstream of the methyl-CpG following DNA chain B, including base-pairs T16T17/A26A25 and T19A20/A23T22, showed a high degree of propeller twist, although the nucleotide sequence is perturbed by base-pair C18/G24. The characteristic high degree of propeller twist, however, was not observed in other regions of the DNA duplex, particularly C/G base-pairs, except for G5/C37, which is unexpectedly highly propeller twisted to 15.1° (Table 4). The major structural effects of a high degree of propeller twist on base-pairing and base-stacking include the following: i) establishment of additional non-Watson-Crick cross-strand diagonal hydrogen bonds and ii) enhanced purine-purine stacking interaction of the adenine bases^31^. Figure 3b shows that on the major groove site of the A/T run, in addition to the conventional Watson-Crick hydrogen bonds, cross-strand bifurcated hydrogen bonds were found to connect purine N6 diagonally to pyrimidine O4 of the opposite strand (N6 of A11 to O4 of T30 and N6 of A28 to O4 of T13). As a result, the bases of the DNA strand are twisted towards its 3′ end, where the N6 of A11 is pushed towards the N4 of T30. Similarly, atom N6 of A28 functions as a bifurcated proton donor to the carbonyl oxygen O4 of T13 and T14 on the opposite strand. Therefore, the AA/TT base-pairs can adopt a zig-zag pattern, as shown in Fig. 3e, whereas this characteristic was not observed in a mixed sequence such as AT/TA or GT/AC, as demonstrated at step A12T13/A29T30. The zig-zag pattern was further extended after the A/T run of the methylated *BDNF*^*ProIII*^helix, except at the base-pair C15/G27. The cross-strand hydrogen bond at base-pair step C15T16/A26G27 does not contribute to the zig-zag configuration because atom C2 of G27 cannot function as a bifurcated proton donor; however, the zig-zag pattern is resumed at base-pair step T16T17/A25A26. This result is consistent with other A/T track double-helix structures reported elsewhere^31^,^33^,^34^. Superimposition of the A/T run of methylated *BDNF*^*ProIII*^ with the Dickerson-Drew dodecamer indeed shows strikingly similar features, particularly the high degree of propeller twist, narrow minor groove width, purine-purine stacking and hydration shells (see below) in the minor groove. The non-coplanarity of the base-pairs as a result of high propeller twist also contributes to the variability of the base-pair buckle, which can be defined as the dihedral angle of the bases along the short base-pair axis after the twisted base-pair has been flattened out by rotating to zero degrees^35^. Table 4 shows that the buckles are highly diverse along the helix, ranging from −11° to 10.4°, with an average of −2.6°. However, the A/T base-pairs displayed an almost planar buckle that averaged to 0.1°. Surprisingly, most of the C/G base-pairs, including methyl-CpG, have buckle greater than ± 6°, with the highest approximately ± 10°. The second structural effect of the high degree of propeller twist is its maximisation of the purine-purine stacking interaction, as observed in the A/T run of methylated *BDNF*^*ProIII*^. This effect correlates to the increased interaction surface between bases on the same DNA strand caused by bases twisting around their longitudinal axes. As shown in Fig. 4a, the adenine bases of the A/T run are stacked on top of each other with their 6-membered rings heavily overlapped, whereas the thymine bases barely overlap at the ring edges and can only make weak van der Waals carbon-carbon contacts between 3–4 atoms of the stacked pyrimidine-pyrimidine. As a result, the DNA sugar-phosphate backbones are brought closer compared with other regions of the DNA, which effectively narrows the minor groove of the A/T base-pairs (Table 3). Similar A/T run characteristics have been observed in A/T rich segments of other DNA double helices^31^,^33^,^34^. However, the base-base compact stacking effect is not observed in the GpG or methyl-CpG of methylated *BDNF*^*ProIII*^ due to the low degree of propeller twist.

### Newly identified water molecules interact with the RTT syndrome-related Glu-137

In this study, a total of 141 water molecules were unambiguously located in the co-crystal structure compared to only 47 water molecules located in the X-ray structure of the MeCP2 MBD in complex with the methylated DNA^27^. The newly identified water molecules provide an insight into the hydration pattern, particularly the ones located in the minor groove of the DNA molecule. Along the 19 base-pairs of double helical DNA, the most hydrated region is located at the major groove of the methyl-CpG dinucleotide. Structural analysis indicates that the DNA-protein contact interface is more hydrated than we previously thought. In addition to the 5 key water molecules that were observed previously (see Fig. 2, Ho *et al*.^27^), several new water molecules have been identified. These water molecules are scattered around the methyl groups and are likely to play stabilising roles and mediate contacts between the key amino acid residues of MeCP2 and the methyl-CpG bases, especially guanines G9 and G34. Among others, the newly identified w116 and w71 are hydrogen bonded to the guanidinium groups of R111 and R133, respectively. The guanine bases of G9 and G34 are gripped in place by the symmetrical arginine fingers of R111 and R133^27^. The side-chain Nε of R133 is connected to the carboxylate group of E137 via a salt bridge, and the side-chain of E137 is surrounded by five mainly hydrophilic groups, including three water molecules (w18, w45 and w71) and the main-chain and side-chain N of R133 (Supplementary Fig. S3). Note that w18 and w71 are newly discovered water molecules. This water network connection prompted us to suspect that the missense mutation E137G, which also leads to RTT syndrome, may disrupt the water network that is required to stabilise the carboxylate group of E137. This possibility is in good agreement with the band shift assay showing that the MBD-DNA binding is reduced by ~80% as a result of the E137G mutation compared to the wild type MBD (Fig. 2a,b). Other newly identified water molecules are distributed around the DNA double helix, specifically along the wall of the DNA minor groove.

### Hydration spines play stabilising role in A/T run geometry

**Figure 5:**
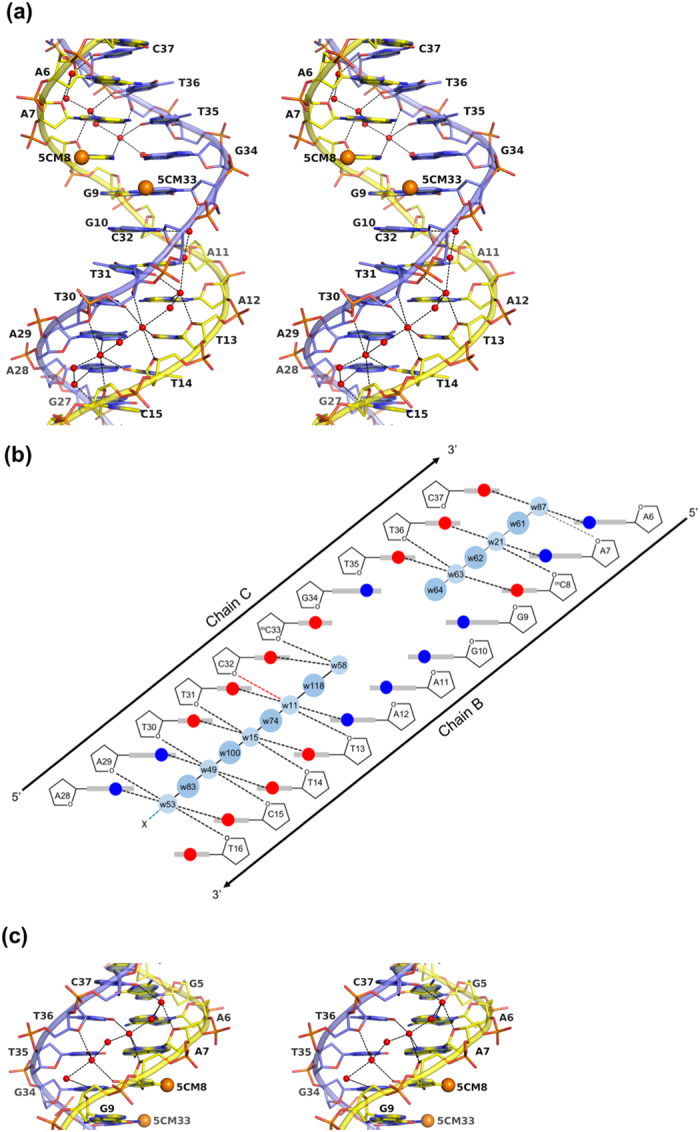
Extensive solvent network in the minor groove of the DNA duplex. (**a**) Stereo view of the DNA duplex from the minor groove site of the A/T run base-pairs. All bases are labelled appropriately. Water molecules are represented by red spheres. All water bridge distances are represented by black dashed lines. Methyl groups are represented by orange spheres. (**b**) Schematic diagram shows the solvent network along the minor groove of the DNA duplex in this study. A string of 9 water molecules runs down the minor groove of the A/T run. Small blue spheres represent inner shell water molecules. Large blue spheres represent outer shell water molecules. Black and red dashed lines connect the inner water molecules to pyrimidine O2, purine N3, and deoxyribose O4′. Black solid and dashed lines show the hydrogen bonding network of the water molecules. (**c**) Stereo view of the DNA duplex from the minor groove site of G5A6A7^m^C8/G34T35T36C37 base-pair steps.

Analysis of the previously solved X-ray structure (PDB code: 3C2I) revealed a short spine of hydration consisting of 3 water molecules in the minor groove of the A/T run. It is unquestionable that the newly identified X-ray structure has provided a more comprehensive account of the hydration pattern around the methylated DNA duplex in complex with the MBD domain. With the 2.2 Å resolution data, the structure shows two hydration spines independently located in the minor groove upstream and downstream of the methyl-CpG dinucleotide following the direction of chain B, as shown in Fig. 5. A hydration spine composed of 9 structured water molecules lies along the wall of the minor groove of the A/T run ^10^GAATT^14^/^28^AATTC^32^ (Fig. 5a). These water molecules can be divided into 2 shells based on their atomic coordination. The inner shell contains water molecules w11, w15, w49 and w53, which can form tetrahedral coordination to 4 different atoms and are positioned symmetrically at the centre between the planes of two base-pairs perpendicular to the helical axis. The outer shell water molecules are w118, w74, w100 and w83, which are located relatively in plane with the respective base-pairs, and each of these water molecules is in principle connected with two inner shell water molecules. The outer shell water molecules are positioned slightly farther from the wall of the minor groove than the inner shell water molecules. As a result, the inner and outer shell water molecules are alternately connected in the A/T run minor groove, forming a zig-zag pattern, as shown in Fig. 5a. Each of the inner shell water molecules is hydrogen bonded to two outer shell water molecules and interacts with the pyrimidine O2 or purine N3 of base *n* and O4′ of the 5-membered deoxyribose ring *n* + 1 on each DNA strand as described previously^36^. This configuration has been observed in diverse examples of A/T track DNA^29^,^37^,^38^,^39^ but has not previously been reported on a methylated DNA in complex with its target protein. All distances from the water oxygen in the minor groove to base N3, O2 and deoxyribose O4′ are listed in Table 5. Strikingly, two of the core inner shell water molecules (w15 and w49) lying in the steps of the A/T run were found to form a hexagonal solvent network with 6 different atoms. At the end of the water string, w53 connects to only one outer shell water molecule, as the subsequent outer shell water molecule in plane with C15/G27 is absent (represented by X in Fig. 5b). At the other end, w11 can only make a pentagonal connection due to a sharp helical rise at step G10A11/T31C32 (Fig. 5a,b), coincident with the kink of the DNA helix, making the deoxyribose O4′ of C32 unreachable by w11 (represented by red dashed line in Fig. 5b). On the other hand, w58 can only connect to 3 atoms, as its position is slightly deviated due in part to the increased minor groove width at base-pair step G9G10/C32^m^C33 (Table 3). In contrast to the base orientation on the major groove of the A/T run, the bases on the minor groove of the A/T run are oriented towards the 5′ direction on its strand so that the displaced O2 of thymine or N3 of adenine can be hydrogen bonded to the first shell water molecules. The extensive water bridging in the minor groove of the A/T run has brought the opposite sugar-phosphate backbones of the A/T run into closer proximity. The narrow minor groove width of the A/T run due to the high degree of propeller twist and purine-purine stacking also significantly affect the solvent network along the minor groove of the A/T run.

Another water string preceding the methyl-CpG dinucleotide (following the chain B sequence) was found in the minor groove of G5A6A7^m^C8/G34T35T36C37 (Fig. 5c). Like the hydration spine in the minor groove of the A/T run, these water molecules can also be divided into inner and outer shells. However, unlike the solvent network made by the inner shell water molecules at the A/T run, the core inner shell water molecules (w21 and w63) of this region can only make pentagonal connections including two outer shell water oxygens, the purine O2 and pyrimidine N3 (or purine O2) and the sugar O4′ of base *n* + 1 (Table 5). Each inner shell water molecule is only hydrogen bonded to one sugar O4′ from either strand of the phosphate-sugar backbone. The inter-phosphate distances of this minor groove average 9.7 Å, which is slightly larger than the A/T run minor groove width (Table 3). Base-pairs G5/C37 and A6/T36 surprisingly display a relatively high degree of propeller twists with an average of 14.5° and significant buckle values ranging from −3.1 to 8.1° (Table 4). Collectively, these factors could be the driving forces that narrow down the minor groove width at this region. However, the base-stacking characteristics of this region are less significant than in the A/T run. The most heavily overlapped are the six-membered rings of G34 and T35 on chain C (Fig. 4b). Other bases are merely overlapped at the ring edge. Overall, the narrowing forces of the minor groove width are weaker than at the A/T run.

Interestingly, along the minor groove from base-pair A6/C37 to T16/A27, the solvent chain is broken at the methyl-CpG dinucleotide step due to a wider minor groove width with an average inter-phosphate distance of 11.7 Å across the methyl-CpG step. Otherwise, the upper and lower hydration spines would join to form a long water string down the DNA double helix (Fig. 5b). Water molecules in the minor groove of the methyl-CpG dinucleotide are not connected to form a water spine, although a few of these water molecules, such as w1, w37, w44 and w85, are hydrogen bonded to either N3 of pyrimidine or O2 of purine. The expansion of the minor groove width could possibly be due to a larger C1′-C1′ distance of 10.9 Å for base-pair mC8/G34 and an average propeller twist of 6.5° (Table 4). These forces geometrically lead to a looser water bridging system and thus allow a more dynamically flexible region for the interaction of the MeCP2 MBD domain.

In summary, the methyl-CpG dinucleotide and A/T run of the B-form DNA attract MeCP2 to its binding target region on chromatin. A cascade of recruitments of transcriptional co-repressors to the promoter region leads to gene silencing as a result of chromatin deacetylation and compaction^40^. The A/T run is stabilised by various forces, including a narrow minor groove, extensive purine-purine stacking, a relatively straight segment of DNA axis and an extensive water network^29^,^31^,^36^. These characteristics are essential to ensure that the B-conformation of the DNA is geometrically stable and will not be unwound or shifted to A- or Z-form. On the other hand, flexibility is allowed in specific regions of the DNA helix, particularly the methyl-CpG dinucleotide, which interacts with the MBD domain. However, natural destabilisation forces such as the insertion of an AT hook into the minor groove could replace water molecules with amino acids, eventually leading to expansion of the minor groove and causing the DNA to bend significantly^30^. It remains unclear how the bending could be affected following geometrical changes to the flanking nucleotides. Although the current X-ray structure of the MeCP2 MBD in complex with the methylated DNA provides a better understanding of the roles of water molecules around the DNA helix, it remains an enigma how the hydration state of the DNA double helix correlates to the MeCP2 functions or chromatin compaction. Further studies on a longer MeCP2 fragment comprising the AT hook in complex with methylated DNA and in association with other co-repressors would provide insights into the roles of water molecules in such complexes.

## Methods

### MeCP2 MBD^A140V^ construction, expression and purification

The MeCP2 MBD^A140V^ and MBD^A140V^-TRD domains were constructed by a site-directed mutagenesis method (Quik-change Site Directed mutagenesis Kit; Stratagene, La Jolla, CA, USA). The recombinant plasmids encoding the native MeCP2 MBD and MBD-TRD coding regions were used as templates to generate mutants MBD^A140V^ and MBD^A140V^-TRD. The point mutation on both constructs was created using a pair of primers (Forward primer: 5′-GCT CTA AAG TGG AGT TGA TTG TGT ACT TCG AAA AGG TAG GCG-3′; Reverse primer: 5′-CGC CTA CCT TTT CGA AGT ACA CAA TCA ACT CCA CTT TAG AGC-3′) that contain the nucleotide substitutions corresponding to A140V. The GC content of the primers is 45.3%, and the melting temperature is 78.4 °C. Amplification of the mutant plasmids was conducted using the thermal profile given by the manufacturer (1 cycle of 95 °C for 30 s; 12 cycles of 95 °C for 30 s, 55 °C for 1 minute and 68 °C for 5.5 minutes; and a final extension of 5 minutes). Following amplification, the template plasmids were digested with *Dpn*I (10 U/μl). The resultant plasmids were introduced into BL21(DE3) *pLysS Escherichia coli* for protein expression.

Expression of all the MBD constructs was induced by adding 1 mM isopropyl β-D-1-thiogalactopyranoside (IPTG) at mid-log phase (OD 600_nm_ = 0.6), and the induction was continued for 6 hours at 30 °C. The cells were harvested by centrifugation (Avanti J-26 XP; Beckman, USA) at 15,000 × *g* at 4 °C. The cell pellet was re-suspended in 20 ml lysis buffer [20 mM Tris-HCl pH 8.0, 500 mM NaCl, 0.1% (v/v) Triton] supplemented with the complete protease-inhibitor mix (Roche) and lysed by ultrasonication (Misonix XL2020; Misonix, USA) for 10 seconds with 15 seconds pulse off for a total of 2 minutes and 30 seconds. Cell debris was removed by centrifugation (Avanti J-26 XP; Beckman, USA) at 48,000 × *g* for 20 minutes at 4 °C. The clarified lysate was filtered with a 0.45 μM Minisart NML syringe filter (Sartorius Stedim) before loading onto a Ni-NTA immobilised metal affinity (IMAC) column (GE healthcare, Sweden) mounted on a fast protein liquid chromatography (FPLC) system (ÄKTA Purifier FPLC system, GE Healthcare, Sweden). The eluted fractions were analysed using SDS-PAGE^41^. All the positive fractions were pooled and concentrated to 1.5 ml before loading onto a HiPrep Sephacryl S-200 HR gel filtration column (GE Healthcare, Sweden) on an FPLC system pre-equilibrated with 20 mM HEPES pH 7.6, 300 mM NaCl. The eluted positive fractions, as analysed with SDS-PAGE, were pooled and concentrated to ~20 mg/ml using a 3 kDa cut-off Amicon Ultra-15 protein concentrator (Millipore; USA).

### Preparation of DNA double helix

A pair of methylated oligonucleotides corresponding to the mouse *BDNF* promoter sequence (methylated *BDNF*^*ProIII*^) were synthesised and purified using reversed-phase HPLC (Oligos Etc.; USA). The oligonucleotides used were 5′-TCT GGA A^m^CG GAA TTC TTC TA-3′ and 5′-ATA GAA GAA TTC ^m^CGT TCC AG-3′. The oligonucleotides were dissolved in TEN buffer (10 mM Tris-HCl pH 8.0, 0.5 mM EDTA, 100 mM NaCl) to a concentration of 1 mM. Both strands were mixed together and annealed by heating to 95 °C for 10 minutes and cooled slowly to room temperature over 3 hours to allow the formation of the DNA double helix.

### Preparation of the MBD-methylated DNA complex

The DNA-protein complex was prepared by mixing MBD^A140V^ and methylated *BDNF*^*ProIII*^ in a ratio of 1:2.6 in CE100 buffer (20 mM HEPES pH 7.9, 100 mM NaCl). The final concentrations of protein and DNA used in crystallisation were 0.2 mM and 0.52 mM, respectively. The mixture was then incubated at room temperature for 30 minutes to allow DNA-protein complex formation.

### Co-crystallisation and structure determination

The MBD^A140V^ in complex with the methylated DNA was co-crystallised using the condition described by Ho *et al*.^27^. The DNA-protein complex co-crystals were grown using the hanging drop vapour diffusion method. Equal volumes (1 μl each) of the DNA-protein complex solution and precipitant solution (30% PEG 2000, 200 mM ammonium acetate, 10 mM Mg acetate and 50 mM, Na cacodylate, pH 6.5) were mixed and equilibrated with the mother liquor at 17 °C in a sealed well for 2–3 days. Crystals were mounted and flash frozen in liquid nitrogen before data collection. Synchrotron X-ray data were collected at wavelength 0.9795 Å at Beamline I02, Diamond Light Source, Didcot, United Kingdom. The DNA-protein co-crystal structure was determined by molecular replacement using an existing X-ray model (PDB code: 3C2I) as the search model^27^. The initial model was refined and rebuilt using *REFMAC 5.7*^42^ and *COOT*^43^. The partially refined model was then submitted to the TLSMD server for TLS (translation/libration/rotation) motion group determination^44^. The multi-group TLS models were further refined with *REFMAC 5.7*^42^. Water molecules of the DNA-protein complex were located with *COOT*^43^. The final model was validated using *PROCHECK*^45^. The DNA geometry of the X-ray structure was analysed by using *3DNA*^46^ and *CURVES*+ 47. A summary of the crystallographic data is shown in Table 1.

### Electrophoretic mobility shift assay (EMSA)

EMSA was conducted at 3 fixed protein concentrations (2 μM, 4 μM and 8 μM), with fixed concentrations of methylated DNA (1.8 μM) and poly(dG-dC).poly(dG-dC) (1.8 μM). The protein and DNA were mixed and incubated in binding buffer (100 mM Tris-HCl pH 7.5, 500 mM KCl, 10 mM DTT) at room temperature for 30 minutes. Sucrose tracking dye [40% (w/v) sucrose, 0.01% (w/v) bromophenol blue and 0.01% (w/v) xynole xylene blue; 2.5 μL] were added to the reaction before electrophoresis was performed on an 8% (w/v) native polyacrylamide gel pre-run for 30 minutes at a constant 50 V, 277 K in Tris-Glycine buffer (0.025 M Tris-HCl pH 8.3, 0.192 M glycine). The gel was electrophoresed for 2 h under the same conditions. The gel was then stained with ethidium bromide for 20 minutes and subsequently destained with dH_2_O for 20 minutes before analysis with a gel documentation system (GelDoc, Bio-Rad Laboratories, Inc., United States) and quantification with the Quantity One 1-D analysis software (Bio-Rad Laboratories, Inc., United States).

### Differential Scanning Calorimetry (DSC)

Differential Scanning Calorimetry (DSC) investigates the thermally induced transitions of a sample from its native to denatured form. In our study, several MeCP2 constructs (wild-type MBD, MBD^A140V^, wild-type MBD-TRD and MBD^A140V^-TRD; 0.06 mM) in complex with methylated DNA (0.156 mM) were analysed by using a VP-DSC microcalorimeter (GE Healthcare). Samples were scanned from 20 to 100 °C with a pre-scan equilibration time of 10 minutes and a scan rate of 1 °C min^−1^. The mid-point melting temperature (*T*_*m*_) was recorded in relative to the excess molar heat capacity [C_p_ (kcal/mole/°C)]. Buffer scan was collected until the baselines became stable. The baseline spectrum was then subtracted from each sample scan using a simple two-state transition substitution and the sample concentrations were normalised to determine the *T*_*m*_. The experimental values were fitted using the Levenberg Marquadt method with the Origin 7.0 software (OriginLab, USA).

### Circular Dichroism (CD)

Circular dichroism (CD) spectra were recorded in the far-UV region using a J-815 JASCO spectrometer (Jasco International Co. Ltd., Hachioji, Tokyo, Japan). MBD constructs, alone or in complex with DNA, were diluted to 5 μM with 100 mM potassium phosphate buffer, pH 6.8 (BioBasic, Canada). The spectra were recorded at 288 K with the spectrometer at wavelengths ranging from 190 to 260 nm, using a bandwidth of 1 nm, through a 1 mm cuvette. The spectrum of the buffer alone was also recorded and subtracted from the protein spectrum. Molar residue ellipticity values were calculated using the spectral analysis software (Jasco Spectra Management software). All spectra were measured in triplicate. The secondary structure content of the MBD constructs was calculated using CONTINLL and reference set 7 on the Dichroweb website^48^.

## Additional Information

**Accession code:** Coordinates have been deposited in Protein Databank under PDB code 5BT2.

**How to cite this article**: Chia, J. Y. *et al*. A/T Run Geometry of B-form DNA is Independent of Bound Methyl-CpG Binding Domain, Cytosine Methylation and Flanking Sequence. *Sci. Rep.* **6**, 31210; doi: 10.1038/srep31210 (2016).

## Supplementary Material

Supplementary Information

## Figures and Tables

**Table 1:** Summary of Data Collection and Refinement Statistics.

Data Collection (Beamline IO2, Didcot)	
Crystal	A140V
Parameters	
Wavelength (Å)	0.9795
Resolution (Å)^a^	48.52–2.18 (2.30–2.18)
Cell bonds (Å)	a = 78.66, b = 53.49, c = 65.78
Cell angles (°)	α = γ = 90°, β = 132.47°
Space group	C2
Unique reflections	10482 (1516)
Completeness (%)	98.9 (98.9)
Average redundancy	3.8 (3.9)
Mean *I*/σ	9.2 (2.6)
R_merge_ (%)^b^	8.8 (80.3)
Refinement	
Resolution range of data used (Å)	48.52–2.18
Reflections used	40175 (5974)
*R* factor (%)^c^	17.5
Free *R* factor (%)^d^	22.7
Average B factors	
Wilson B factor (Å^2^)	61.8
Protein (Chain A)	50.8
DNA (Chain B)	48.9
DNA (Chain C)	52.3
Water molecules	69.4
Number of protein molecules in asymmetric unit	1
Total number of non-hydrogen atoms	
Protein	572
Non-protein	407
Solvent	409
RMSD from standard values	
Bonds (Å)	0.0113
Angles (°)	1.8228
Ramachandran plot^e^	
Residues in favoured region (%)	93.1
Residues in allowed region (%)	6.9
Residues in disallowed region (%)	0.0

^a^Values in parentheses are for the highest resolution shell.

^b^*R*_*merge*_ = 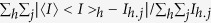, where 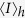 is the mean intensity of symmetry-equivalent reflections.

^c^R factor = 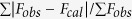, where F_obs_ and F_cal_ are the observed and calculated structure factor amplitudes, respectively.

^d^Free R factor value was calculated for R factor using only an unrefined subset of reflections data (5%).

^e^Ramachandran plot was calculated using *PROCHECK*^49^.

**Table 2:** Circular dichroism and differential scanning calorimetry analyses.

MeCP2 constructs	Secondary structure analysis (%) ± S.D			*T*_*m*_ (°C) ± S.D
	α-Helices	β-Strands	Flexible regions	
WT MBD + DNA	20.5 ± 0.3	49.8 ± 0.3	29.8 ± 0	50.2 ± 0.2
WT MBD	14.7 ± 0.2	44.9 ± 0.2	40.4 ± 0.3	N.D
MBD^A140V^ + DNA	16.3 ± 0.3	52.9 ± 0.3	30.9 ± 0.5	43.2 ± 0.2
MBD^A140V^	11.7 ± 0.2	49.4 ± 0.2	38.9 ± 0.2	N.D
WT MBD-TRD + DNA	15.7 ± 0.5	51.8 ± 0.4	32.5 ± 0.5	46.7 ± 0.2
WT MBD-TRD	8 ± 0.3	54.4 ± 0.3	37.7 ± 0.3	N.D
MBD^A140V^-TRD + DNA	15.5 ± 0.5	51.8 ± 0	32.5 ± 0	39.8 ± 0.2
MBD^A140V^-TRD	3.5 ± 0.2	61.4 ± 0.3	35.1 ± 0.3	N.D

Circular dichroism was performed to analyse the secondary structures of wild-type and mutant forms of the MBD and MBD-TRD domains of MeCP2 in complex with methylated DNA. The secondary structure contents of the MBD and MBD-TRD domain complexes were based on CONTINLL Deconvolution of CD data. Melting temperatures [*T*_m_] of the wild type and mutants of MeCP2 MBD and MBD-TRD in complex with methylated DNA were measured by differential scanning calorimetry (DSC). Analyses were performed in triplicate, and standard deviations (S.D) were calculated accordingly. N.D; not determined.

**Table 3:** Local base-pair step parameters.

Base-pair step	Minor groove width (Å)	Major groove width (Å)	Slide (Å)	Roll (°)	Tilt (°)	Rise per base (Å)
C2 ≡ G40						
**| |**	—	—	−0.2	−1.3	1.4	3.0
T3 = A39						
**| |**	—	—	0.9	4.8	0.5	3.2
G4 ≡ C38						
**| |**	12.5	18.1	0.3	3.8	−2.4	3.4
G5 ≡ C37						
**| |**	10.9	17.3	−0.4	1.9	−1.7	3.5
A6 = T36						
**| |**	9.7	16.4	−0.4	−0.7	−2.8	3.2
A7 = G35						
**| |**	9.7	17.5	−0.6	−3.7	0.9	3.4
^m^C8 = T34						
| |	11.2	18.9	0.3	2.3	1.8	3.4
G9 ≡ ^m^C33						
**| |**	12.3	17.9	0.5	3.3	0.1	3.1
G10 ≡ C32						
**| |**	11.6	16.5	1.1	1.7	−3.5	3.3
A11 = T31						
| |	9.9	18.8	0.0	−2.0	1.8	3.3
A12 = T30						
| |	8.7	17.4	−0.6	0.2	−1.9	3.2
T13 = A29						
| |	9.0	20.0	−0.5	−2.9	0.6	3.2
T14 = A28						
**| |**	9.3	19.8	−0.5	−1.0	−1.2	3.5
C15 ≡ G27						
**| |**	9.6	17.2	0.1	−1.7	1.3	3.1
T16 = A26						
**| |**	10.3	18.1	0.3	−2.7	−1.5	3.3
T17 = A25						
**| |**	10.2	16.8	1.2	−2.1	0.4	3.4
C18 ≡ G24						
**| |**	—	—	0.7	−3.0	0.0	3.3
T19 = A23						
**| |**	—	—	1.6	−1.3	3.3	3.4
A20 = T22						
**Average**	**10.4**	**17.9**	**0.2**	**−0.2**	**−0.2**	**3.3**

The minor and major groove widths were measured considering the directions of the sugar-phosphate backbones. A useful measurement of the inter-phosphate distance across the major opening is obtained by subtracting 5.8 Å of the van der Waals radii of two phosphate groups from the calculated values. “=” and “≡” represent the conventional Watson-Crick hydrogen bonds between adenine-thymine and cytosine:guanine base-pairs, respectively.

**Table 4:** Local base-pair parameters.

Base-pair	Propeller twist (°)	Buckle (°)	C1′-C1′ (Å)
C2/G40	−5.3	−11.0	10.7
T3/A39	−3.1	1.6	10.6
G4/C38	−4.7	10.4	10.6
G5/C37	−15.1	8.1	10.6
A6/T36	−13.8	−3.1	10.3
A7/T35	−8.7	−4.2	10.4
m5C8/G34	−6.9	−6.0	10.9
G9/m5C33	−6.1	−9.1	10.5
G10/C32	−3.7	0.2	10.6
A11/T31	−18.2	3.4	10.6
A12/T30	−13.4	−2.0	10.5
T13/A29	−11.7	0.2	10.5
T14/A28	−13.1	0.9	10.5
C15/G27	−9.7	−9.6	10.8
T16/A26	−12.6	−1.7	10.4
T17/A25	−17.3	−6.1	10.8
C18/G24	−4.1	−7.8	10.5
T19/A23	−21.8	−6.9	10.9
A20/T22	−10.3	−6.1	10.6
**Average**	**−10.5**	**−2.6**	**10.6**

**Table 5:** Inner shell water bridges along the floor of the DNA minor groove.

Water molecule	Nucleotide	Atom	Distance (Å)
w87	A6	N3	2.8
	A7	Sugar O4′	2.9
	C37	O2	3.0
w21	A7	N3	2.6
	mC8	Sugar O4′	3.1
	T36	O2	2.9
w63	mC8	O2	2.9
	T35	O2	3.1
	T36	Sugar O4′	3.2
w58	C32	O2	2.7
	mC33	Sugar O4′	2.7
w11	A12	N3	2.9
	T13	Sugar O4′	3.1
	T31	O2	2.8
w15	T13	O2	2.5
	T14	Sugar O4′	2.9
	T30	O2	2.8
	T31	Sugar O4′	3.5
w49	T14	O2	2.7
	C15	Sugar O4′	3.2
	A29	N3	2.8
	T30	Sugar O4′	3.2
w53	C15	O2	2.7
	T16	Sugar O4′	3.5
	A28	N3	2.8
	A29	Sugar O4′	3.3
