# Supplementary material for: A/T Run Geometry of B-form DNA Is Independent of Bound Methyl-CpG Binding Domain, Cytosine Methylation and Flanking Sequence
Source: Sci Rep. 2016 Aug 9;6:31210. doi: 10.1038/srep31210 (PMC4977486; doi:10.1038/srep31210)
Supplement: Supplementary Information [file srep31210-s1.pdf]

A/T Run Geometry of B-form DNA Is Independent of Bound Methyl-CpG Binding Domain, Cytosine Methylation and Flanking Sequence

JYH YEA, CHIA[1]; WEN SIANG, TAN[2,4]; CHYAN LEONG, NG[5]; NIEN-JEN, HU[6]; HOOI LING, FOO[3,4] and KOK LIAN, HO[1*]

Supplementary Figure 1: Circular dichroism analysis

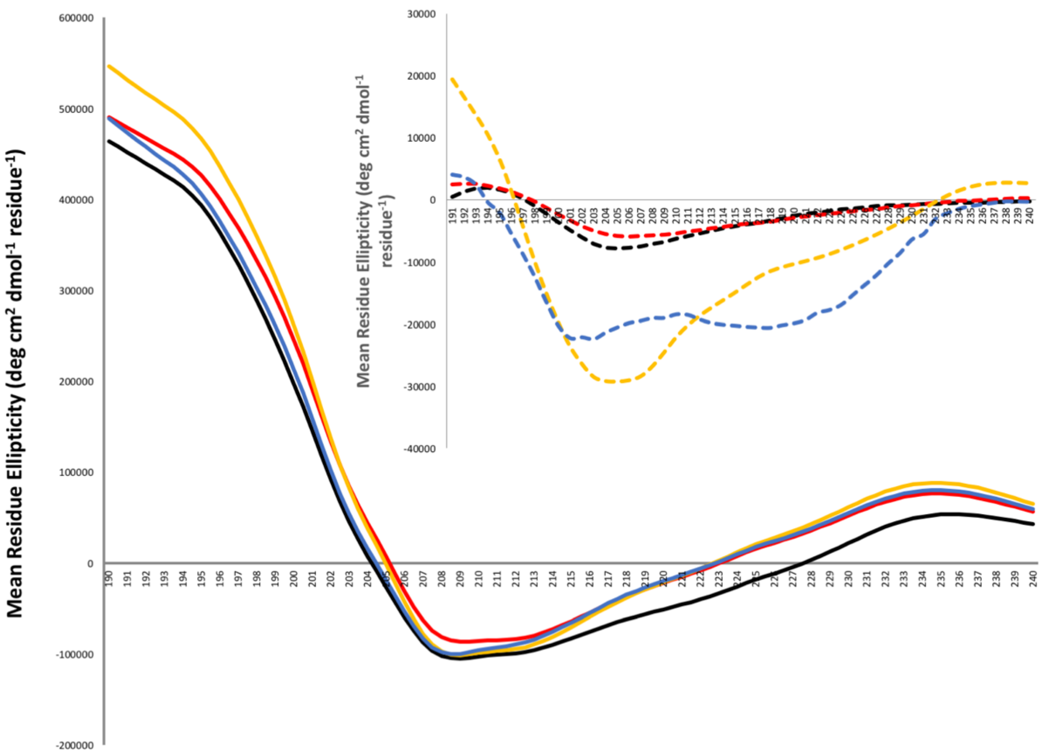

Circular dichroism (CD) spectra of wild type and mutant MeCP2 constructs in complex with methylated DNA were scanned from wavelength 190 nm to 240 nm. The spectra of wild type MBD (black line), MBD^{A140V} (red line), wild type MBD-TRD (yellow line) and MBD^{A140V}-TRD (blue line) in complex with methylated DNA are shown. Inset shows the CD spectra of the wild type MBD (black dotted lines) and MBD^{A140V} (red dotted line), wild type MBD-TRD (yellow dotted line) and MBD^{A140V}-TRD (blue dotted line) alone.

Supplementary Figure S2: Differential Scanning Calorimetry analysis

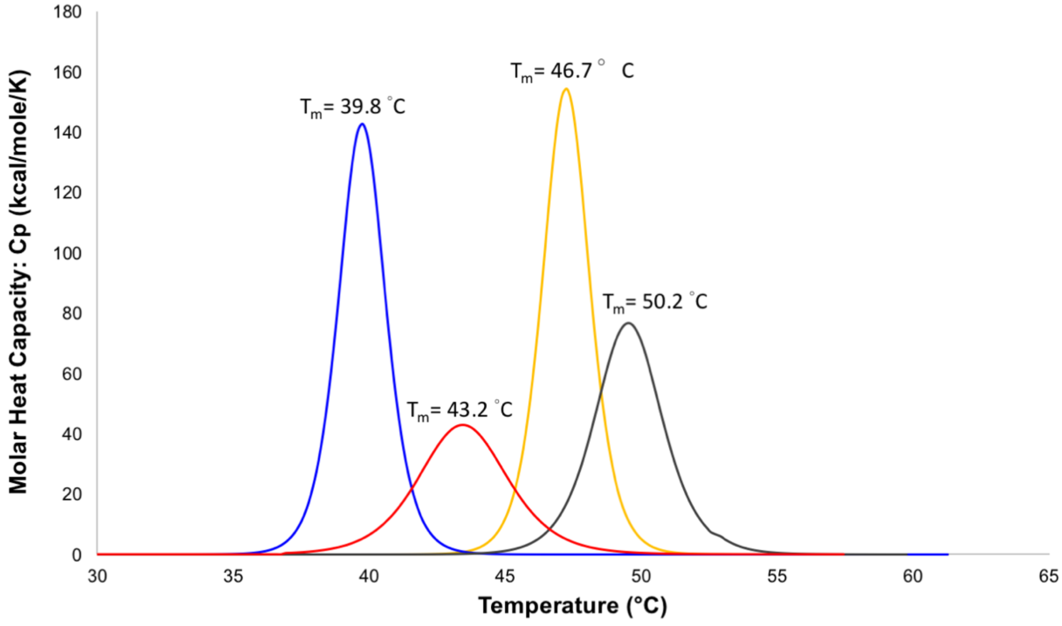

The diagram shows the first derivative plot of the data with Gaussian fits. Top of the transition indicates the melting temperature (T_m). Thermal unfolding parameters of the complex of methylated DNA with MeCP2 constructs were scanned from 0 to 100 °C. For clarity purposes, only thermal profiles from 30 to 65 °C are shown. The thermal profile of wild type MBD, MBD^{A140V}, wild type MBD-TRD and MBD^{A140V}-TRD are represented by black, red, yellow and blue lines, respectively. X- and Y-axes represent the molar heat capacity and the melting temperature, respectively.

Supplementary Figure S3: E137 is stabilised by water molecules and amide groups

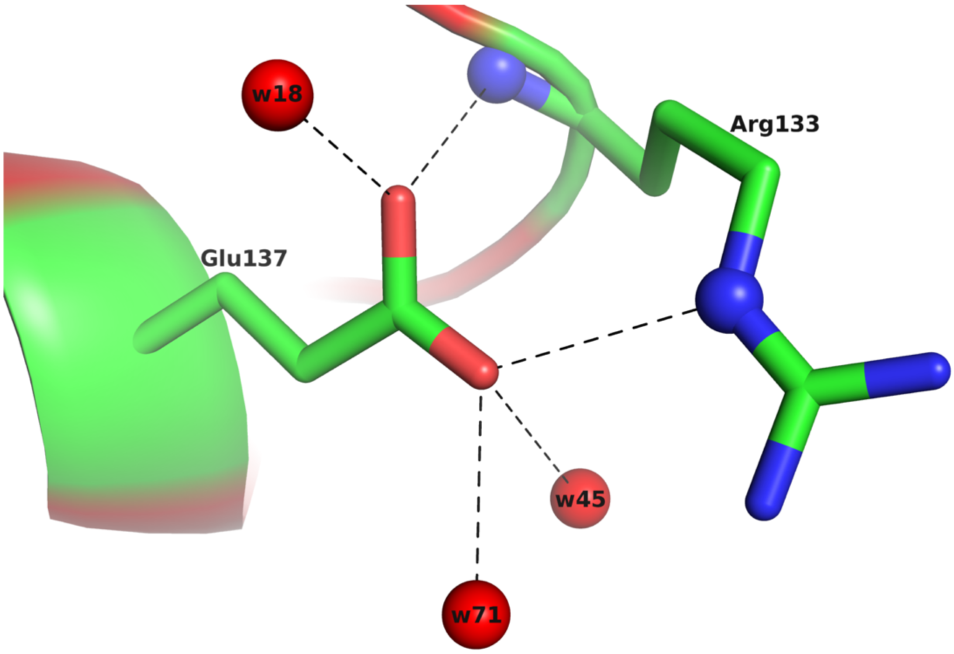

The carboxylate group of E137 is stabilised by water molecules w18, w45 and w71, and the main-chain and side-chain amide of R133. Black dashed lines show all distances below 3.5 Å. Water molecules are shown as red spheres. E137 and R133 are indicated. Diagram is simplified for clarity.
